# Dietary tryptophan intervention counteracts stress-induced transcriptional changes in a teleost fish HPI axis during inflammation

**DOI:** 10.1038/s41598-024-57761-0

**Published:** 2024-03-28

**Authors:** Diogo Peixoto, Inês Carvalho, Marina Machado, Cláudia Aragão, Benjamín Costas, Rita Azeredo

**Affiliations:** 1 CIIMAR-Centro Interdisciplinar de Investigação Marinha e Ambiental, Av. General Norton de Matos s/n, 4450-208 Matosinhos, Portugal; 2 ICBAS-Instituto de Ciências Biomédicas Abel Salazar, Universidade do Porto, Porto, Portugal; 3 Departamento de Biología Facultad de Ciencias del Mar y Ambientales, Instituto Universitario de Investigación Marina (INMAR), Campus de Excelencia Internacional del Mar (CEIMAR), Universidad de Cádiz, Puerto Real, Spain; 4 Centro de Ciências do Mar (CCMAR), Faro, Portugal; 5 Universidade do Algarve, Faro, Portugal

**Keywords:** Stress response, Fish physiology, Molecular immunology, Nutritional immunology, Immune-neuroendocrine interaction, Acute inflammation, Marine biology

## Abstract

Immune nutrition is currently used to enhance fish health by incorporating functional ingredients into aquafeeds. This study aimed to investigate the connections between tryptophan nutrition and the network that regulates the communication pathways between neuroendocrine and immune systems in European seabass (*Dicentrarchus labrax*). When tryptophan was supplemented in the diet of unstressed fish, it induced changes in the hypothalamic-pituitary-interrenal axis response to stress. Tryptophan-mediated effects were observed in the expression of anti-inflammatory cytokines and glucocorticoid receptors. Tryptophan supplementation decreased pro-opiomelanocortin b-like levels, that are related with adrenocorticotropic hormone and cortisol secretion. When stressed fish fed a tryptophan-supplemented diet were subjected to an inflammatory stimulus, plasma cortisol levels decreased and the expression of genes involved in the neuroendocrine response was altered. Modulatory effects of tryptophan dietary intervention on molecular patterns seem to be mediated by altered patterns in serotonergic activity.

## Introduction

Fish production, similar to any other animal-intensive production system, entails stress that is induced by transport, crowding conditions, overfeeding, suboptimal water quality, and handling procedures such as vaccination, compromising fish welfare^1–3^. Stressful rearing conditions are described as affecting flesh quality (e.g., lower muscle pH and faster meat quality deterioration before slaughter), while decreasing growth rates and rendering fish more prone to pathologies^4–12^. Since quality and welfare issues are intrinsically linked, the implications of stressful environments have gradually increased awareness of fish consumers^8,13^.

Currently, immune nutrition is used to maintain and improve animal health by incorporating functional ingredients in aquafeeds that stimulate or modulate fish immune system, as a complementary prophylactic strategy, for example, to vaccination in aquaculture. These ingredients are selected based on their target immune properties and feeds are formulated not to compromise nutritional requirements for basal physiological needs. Tryptophan is an essential amino acid that is transported through the brain blood barrier in competition with other large neutral amino acids (i.e., valine, isoleucine, leucine, tyrosine, phenylalanine and methionine). In a reaction involving tryptophan hydroxylase (TPH), tryptophan is converted into the neurotransmitter serotonin (5-hydroxytryptamine; 5-HT) that modulates the neuroendocrine response. Thereby, tryptophan mediates modulatory effects on both stress and behavioural responses^1,10,14–16^.

The nervous, endocrine, and immune systems are closely related, rely on each other, and are integrated into a complex bi-directional network of signalling molecules, receptors, and regulatory mechanisms. Indeed, molecules from the immune system such as cytokines can regulate endocrine activity^12^. On the other hand, certain cells of the nervous and endocrine systems share the ability to produce specific proteins that may work as immune modulators or metabolic regulators. A practical example of such an integrated response is the effects of stress on the immune system, mostly mediated by the stress hormone cortisol (immunosuppressive hormone). In fish, the stress response is coordinated by the hypothalamus-pituitary-interrenal axis (HPI), initiated with the release of hypothalamic corticotropin-releasing hormone (CRH) and culminates in cortisol secretion by the interrenal cells of the head-kidney^3,17,18^. High levels of glucocorticoids (such as cortisol) suppress humoral factors involved in the inflammatory response (such as cytokine production of immune cells), inhibit leukocyte mobilization to inflammatory sites, and overall reduce circulating leucocytes and lymphocytes^3,19–24^.

Since TPH is not saturated at tryptophan physiological concentrations, 5-HT production can be promoted with tryptophan supplementation^14^. Indeed, some studies highlighted tryptophan modulatory effects on teleost HPI axis. Lepage et al.^25,26^, observed that rainbow trout (*Oncorhynchus mykiss*) fed a tryptophan-supplemented diet for 1 week (up to 4× the requirement level), presented a significant attenuation of stress-induced elevation of plasma cortisol compared to those fed a control diet. Azeredo et al.^27^, reported an immune suppressive effect of tryptophan-supplemented diets (2× the requirement level) in fish undergoing an acute inflammatory response, along with higher production of brain monoamines and cortisol levels. Interestingly, a different, recently published approach to the hereby presented experiment, reported several changes in the inflammatory response of *Photobacterium damselae* subsp. *piscicida* (*Phdp*)-infected seabass when fish were given a tryptophan surplus for 15 days, such as upregulation of immune-related genes, the inversion of the stress-induced T-cell suppression and an impairment of bacterial injection-induced cortisol production^28^. Moreover, chronically stressed Senegalese sole (i.e., held at high stocking density) fed a tryptophan-supplemented diet (4× the requirement level) for 38 days presented an alleviated stress response (lower cortisol levels and downregulation of pro-opiomelanocortin gene expression) that enabled a more efficient immune response and increased disease resistance^29^. Moreover, there is a significant number of studies focused on the effect of high stocking density on fish physiological responses, but, to the best of the authors’ knowledge, no data have been published regarding European seabass or any other species reared under space confinement conditions.

The present study aimed to explore the fundamental links between tryptophan nutrition and the network that regulates the bi-directional pathways between neuroendocrine and immune systems in European seabass (*Dicentrarchus labrax*). Hence, a special focus is given to the expression patterns of both the brain and head-kidney of seabass reared under chronic stressful conditions by space confinement and subsequently exposed to an infection episode.

Outcomes generated from this study are expected to provide a better understanding of tryptophan’s modulatory role in European seabass immune and neuroendocrine responses. This opens a new avenue for research in nutritional immunology within aquaculture.

## Material and methods

### Diets composition

The experimental diets were formulated and manufactured by Sparos Lda. (Olhão, Portugal). The control diet (CTRL) was formulated to fulfil the known indispensable amino acid requirements of European seabass^30^. Then, the CTRL diet was supplemented with 0.3% l-tryptophan (dry matter; TRP), at the expense of wheat meal. Chemical and amino acid composition of experimental diets are presented in Tables 1 and 2, respectively.

**Table 1:** Ingredients and chemical composition of the experimental diets.

Ingredients (%)	CTRL	TRP
CPSP 90^a^	5.00	5.00
Fish gelatin^b^	2.00	2.00
Soy protein concentrate^c^	25.00	25.00
Pea protein concentrate^d^	6.00	6.00
Wheat gluten^e^	10.00	10.00
Corn gluten meal^f^	15.00	15.00
Wheat meal^g^	15.80	15.50
Vitamins and minerals premix^h^	1.00	1.00
Antioxidant^i^	0.20	0.20
Sodium propionate^j^	0.10	0.10
MCP^k^	3.00	3.00
l-Lysine HCl 99%^l^	0.60	0.60
l-Tryptophan^m^	0.00	0.30
dl-Methionine^n^	0.20	0.20
Soy lecithin^o^	1.00	1.00
Fish oil^p^	15.10	15.10
Total	100.00	100.00
Proximate analysis (% dry weight)		
Crude protein	45.70	46.00
Crude fat	18.00	18.00
Fiber	1.70	1.70
Starch	13.40	13.20
Ash	6.80	6.80
Energy (MJ kg^−1^)	21.90	21.90

^a^CPSP 90: 82.6% crude protein (CP), 9.6% crude fat (CF), Sopropêche, France.

^b^Fish gelatin: 88% CP, 0.1% CF, LAPI Gelatine SPA, Italy.

^c^Soycomil P: 63% CP, 0.8% CF, ADM, The Netherlands.

^d^NUTRALYS F85F: 78% CP, 1% CF, ROQUETTE Frères.

^e^VITAL: 83.7% CP, 1.6% CF, ROQUETTE Frères, France.

^f^Corn gluten meal: 61% CP, 6% CF, COPAM, Portugal.

^g^Wheat meal: 10.2% CP; 1.2% CF, Casa Lanchinha, Portugal.

^h^PREMIX Lda, Portugal: Vitamins (IU or mg kg^−1^ diet): dl-alpha tocopherol acetate, 100 mg; sodium menadione bisulphate, 25 mg; retinyl acetate, 20,000 IU; dl-cholecalciferol, 2000 IU; thiamin, 30 mg; riboflavin, 30 mg; pyridoxine, 20 mg; cyanocobalamin, 0.1 mg; nicotinic acid, 200 mg; folic acid, 15 mg; ascorbic acid, 500 mg; inositol, 500 mg; biotin, 3 mg; calcium panthotenate, 100 mg; choline chloride, 1000 mg, betaine, 500 mg. Minerals (g or mg kg^-1^ diet): copper sulphate, 9 mg; ferric sulphate, 6 mg; potassium iodide, 0.5 mg; manganese oxide, 9.6 mg; sodium selenite, 0.01 mg; zinc sulphate, 7.5 mg; sodium chloride, 400 mg; excipient wheat middlings.

^i^Paramega PX, Kemin Europe NV, Belgium.

^j^PREMIX Lda., Portugal.

^k^Monocalcium phosphate: 22% phosphorus, 16% calcium, Fosfitalia, Italy.

^l^l-Lysine HCl 99%, Ajinomoto Eurolysine SAS, France.

^m^l-Tryptophan 98%, Ajinomoto Eurolysine SAS, France.

^n^dl-Methionine for Aquaculture: 99% Methionine, Evonik Nutrition & Care GmbH, German.

^o^Lecico P700IPM, LECICO GmbH, Germany.

^p^SAVINOR UTS, Portugal.

**Table 2:** Amino acid composition of experimental diets.

Amino acids (% dry weight)	CTRL	TRP
Arginine	4.0	4.1
Histidine	1.2	1.2
Lysine	3.0	3.0
Threonine	1.8	1.9
Isoleucine	2.1	2.1
Leucine	4.2	4.3
Valine	2.3	2.3
Tryptophan	0.2	0.4
Methionine	1.1	1.2
Phenylalanine	3.0	3.2
Cysteine	0.4	0.4
Tyrosine	2.6	2.8
Aspartic acid	3.4	3.2
Glutamic acid	9.7	9.1
Alanine	2.4	2.5
Glycine	2.7	2.7
Proline	3.7	3.6
Serine	2.6	2.6

### *Phdp* inoculum preparation

The inflammatory bacterial challenge was performed using *Photobacterium damselae piscicida* (*Phdp*) strain PP3, isolated from yellowtail (*Seriola quinqueradiata*; Japan) by Doctor Andrew C. Barnes (Marine Laboratory, Aberdeen, UK), following the methodology described in Machado et al.^28^.

### Experimental design

**Figure 1:**
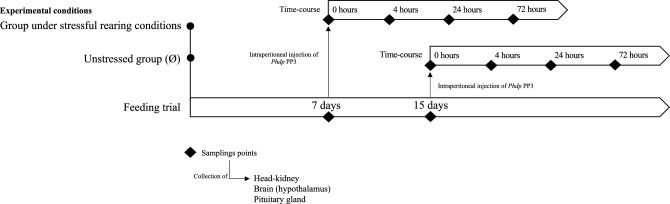
Experimental setup of dietary tryptophan supplementation during acute inflammation under stressful conditions.

European seabass juveniles (12.02 ± 2.77 g) were randomly distributed in four independent recirculating seawater systems with 8 tanks each (52 L, 26 cm height, n = 22 *per* tank) with a density of 5 kg m^−3^ (temperature 20.0 ± 0.5 °C; salinity 32 ‰; photoperiod 10:14 h dark:light). Two feeding periods were tested in parallel (7 and 15 days; two systems for each feeding period). In a complete randomized design, the two dietary treatments were evaluated in quadruplicate tanks of each system. Fish were fed these diets twice a day with a daily average ration of 2% of body weight. In both feeding trials, by lowering the water level in one of the systems, fish were kept under stressful conditions induced by space confinement (i.e., 8 tanks with a density of 10 kg m^−3^ in 26 L and 13 cm height) and consequently, under stressful conditions. The lower density groups served as control (Ø). At the end of each feeding period, 8 fish *per* treatment were euthanized by an overdose of 2-phenoxyethanol and the blood, hypothalamus, pituitary gland and head-kidney samples were collected. As no further stimulation was inflicted on these fish, these first sampled groups were considered undisturbed (0 h, Fig. 1). The remaining fish were intraperitoneally injected (i.p.) with 100 µL of *Phdp* (5 × 10^7^ cfu mL^−1^) and moved to a new system with the same dimensions to avoid horizontal infection with other groups (temperature 24.0 ± 0.5 °C; salinity 32 ‰; photoperiod 10:14 h dark:light) and similarly sampled at 4-, 24- and 72-h post-infection. The temperature increased 2 °C *per* day until 24 °C to avoid a new stressful factor. No mortality was observed during the trial. The experiments were approved by the Animal Welfare Committee of the Interdisciplinary Centre of Marine and Environmental Research and carried out in a registered installation (N16091.UDER). All experiments were performed by trained scientists (following FELASA category C recommendations) in full compliance with national rules, following both the European Directive 2010/63/EU of the European Parliament and the European Union Council on the protection of animals used for scientific purposes, and the relevant ARRIVE guidelines.

### Blood collection and assessment of plasma cortisol levels

Blood was withdrawn from the caudal vein using heparinized syringes and centrifuged at 10,000×*g* 10 min at 4 °C. Plasma was collected, frozen in dry ice, and stored at − 80 °C for later evaluation of cortisol levels. Cortisol was assessed using an ELISA kit (IBL International Gmbh, Hamburg, Germany) following manufacturer’s instructions and according to Azeredo et al.^14^. Results regarding plasma cortisol levels from fish fed dietary treatments for 15 days are publish elsewhere^28^.

### Gene expression analysis (hypothalamus, pituitary gland and head-kidney)

Hypothalamus and head-kidney were individually processed for total RNA extraction using the NZY Total RNA Isolation kit (NZYTech) following manufacturer’s instructions. Pituitary glands’ total RNA was extracted using NucleoSpin RNA XS kit (Macherey Nagel) also following manufacturer’s instructions. For all tissues, first-strand cDNA was synthesized with NZY First-Strand cDNA Synthesis Kit (NZYTech). DNA amplification was carried out with specific primers for genes that have been selected for their involvement in immune response and oxidative stress. Sequences encoding European seabass were identified after carrying out a search in the databases, such as databases v1.0c seabass genome and primers were designed with NCBI Primer Designing Tool according to known qPCR restrictions (amplicon size, Tm difference between primers, GC content and self-dimer or cross-dimer formation). Accession number, efficiency values, annealing temperature, product length and primers sequences used in the hypothalamus, pituitary gland and head-kidney gene expression are presented in Table 3. Real-time quantitative PCR was carried out in a CFX384 Touch Real-Time PCR Detection System (Biorad), using 4.4 μl of diluted cDNA mixed with 5 μl of iTaq Universal SYBR green supermix (BioRad) and 0.3 μl (10 μM) of each specific primer in a final volume of 10 μl. The standard cycling conditions were initial denaturation for 10 min at 95 °C, followed by 40 cycles of two steps (95 °C denaturation for 15 s followed by primer annealing temperature (Table 3) for 1 min), 95 °C for 1 min followed by 35 s at the annealing temperature, and finally, 95 °C for 15 s. All reactions were carried out as technical duplicates. Melting curve analysis was also performed to verify that no primer dimers were amplified. For all tissues, the expression of the target genes was normalized using the expression of European seabass elongation factor 1-α (*ef1α*) and 40s ribosomal protein (*40s*).

**Table 3:** Oligonucleotide sequences used to evaluate relative mRNA abundance of genes in the hypothalamus, pituitary gland and head-kidney by RT-qPCR, including forward (F) and reverse (R) primers, GenBank ID (NCBI), efficiencies (E) of qPCR reactions and annealing temperature (Ta).

Gene	Acronym	GenBank ID	E	Ta (ºC)	Primer sequence (5′–3′)
Hypothalamus					
Elongation factor 1-α	*ef1α*	AJ866727.1	2.31	60	F: AACTTCAACGCCCAGGTCAT R: CTTCTTGCCAGAACGACGGT
40s ribosomal protein	*40s*	HE978789.1	2.23	60	F: TGATTGTGACAGACCCTCGTG R: CACAGAGCAATGGTGGGGAT
Interleukin 1 β	*il1β*	AJ269472.1	2.04	60	F: AGCGACATGGTGCGATTTCT R: CTCCTCTGCTGTGCTGATGT
Interleukin 6	*il6*	AM490062.1	2.04	55	F: AGGCACAGAGAACACGTCAAA R: AAAAGGGTCAGGGCTGTCG
Interleukin 10	*il10*	AM268529.1	2.06	55	F: ACCCCGTTCGCTTGCCA R: CATCTGGTGACATCACTC
Glucocorticoid receptor 1	*gr1*	AY619996.1	2.13	60	F: AAATCTGCCTGGTGTGTTCC R: TGCCCTTTCACTGCTCTCTT
Glucocorticoid receptor 2	*gr2*	AY549305.1	2.18	60	F: CTTCTACAGCACCAGCACCA R: TCTCCTGTTTGACCACACCA
Corticotropin releasing hormone-binding protein	*crhbp*	MG832822.1	2.09	60	F: TGTCATCTCCCAGTCACCAG R: GCCATTTCCTCCAAGCAAC
Corticotropin releasing hormone	*crh*	JF274994.1	1.97	60	F: AACCCAAAACTCCCAGCAG R: TGTTCCCAACTTTCCCTTGT
5-hydroxytryptamine (serotonin) receptor 1A β-like	*htr1aβ*	DLAgn_00119560^a^	2.33	60	F: GGAGCGTAAAACGGTGAAAA R: TGGGGTTGAGGAGAGAGTTG
Macrophage colony-stimulating factor 1 receptor 1	*mcsf1r1*	DLAgn_00109630^a^	2.26	61	F: ATGTCCCAACCAGACTTTGC R: GGCTCATCACACACTTCACC
Tumor necrosis factor-alpha	*tnfα*	DQ070246.1	2.03	55	F: AGCCACAGGATCTGGAGCTA R: GTCCGCTTCTGTAGCTGTCC
Transforming growth factor-beta	*tgfβ*	AM421619.1	2.04	55	F: ACCTACATCTGGAACGCTGA R: TGTTGCCTGCCCACATAGTAG
Tryptophan 5-hydroxylase-like	*tph1α*	DLAgn_00154580^a^	1.98	60	F: CGCATAGACTTCACAACAGAGG R: CAGCAGAGGGAGGTTCTTCA
μ opioid receptor	*muor*	DLAgn_00015310^a^	2.04	60	F: GTCACCAGCACCCTACCATT R: CGAGGAGAGAATCCAGTTGC
κ-type opioid receptor-like 2	*kor2*	DLAgn_00007470^a^	1.96	60	F: TCTGGTGCTTGTGGTAGTCG R: TGGCAGTCTCTGTGTCCTTG
δ-opioid receptor	*dor2*	DLAgn_00062690^a^	2.12	60	F: CGCTTCTCGGTCTCCATAACT R: GGTCTCATTACTACTTGAAG
Opioid growth factor receptor 2	*ogfr2*	DLAgn_00128530^a^	2.23	60	F: GTTGGGAATGGAGATGGAAA R: GCTTCAGATTTTGGCTCAGG
Pituitary gland					
Elongation factor 1-α	*ef1α*	AJ866727.1	2.35	60	F: AACTTCAACGCCCAGGTCAT R: CTTCTTGCCAGAACGACGGT
40s ribosomal protein	*40s*	HE978789.1	2.48	60	F: TGATTGTGACAGACCCTCGTG R: CACAGAGCAATGGTGGGGAT
Interleukin 1 β	*il1β*	AJ269472.1	2.17	60	F: AGCGACATGGTGCGATTTCT R: CTCCTCTGCTGTGCTGATGT
Glucocorticoid receptor 1	*gr1*	AY619996.1	2.24	60	F: AAATCTGCCTGGTGTGTTCC R: TGCCCTTTCACTGCTCTCTT
5-hydroxytryptamine (serotonin) receptor 2a	*htr2a*	DLAgn_00222310^a^	2.19	60	F: CCTCTGACCTCTGTCCCATC R: ACTGAAATCGTCCACACTGC
Tryptophan 5-hydroxylase-like	*tph1α*	DLAgn_00154580^a^	2.01	60	F: CGCATAGACTTCACAACAGAGG R: CAGCAGAGGGAGGTTCTTCA
Pro-opiomelanocortin a-like	*pomca*	AY691808.1	2.28	60	F: TCTTCCTCCTCCTCTCCACA R: CGCCTTCTCATCTCTTCAGG
Pro-opiomelanocortin b-like	*pomcb*	DLAgn_00069720^a^	1.97	60	F: GGTTGTTAGTGGTGGTGATGG R: GTCCTTGTTGCTCAGGTCGT
Head-kidney					
Elongation factor 1-α	*ef1α*	AJ866727.1	2.35	57	F: AACTTCAACGCCCAGGTCAT R: CTTCTTGCCAGAACGACGGT
40s ribosomal protein	*40s*	HE978789.1	2.48	60	F: TGATTGTGACAGACCCTCGTG R: CACAGAGCAATGGTGGGGAT
Glucocorticoid receptor 1	*gr1*	AY619996.1	2.24	60	F: AAATCTGCCTGGTGTGTTCC R: TGCCCTTTCACTGCTCTCTT
Melanocortin 2 receptor	*mc2r*	DLAgn_00065140^a^	2.01	60	F: GAGGGCAAGGGGAGCATTTA R: GACGGGCAGATGGCAGTTAT
Indoleamine-dioxygenase 2	*ido2*	DLAgn_00014730^a^	2.08	55	F: TGAAGGTGTGAGCAATGAGC R: CAAAGCACTGAATGGCTGAA

^a^Sequences obtained from databases dicLab v1.0c seabass genome.

### Data analysis

All results are expressed as mean ± standard deviation (SD). Shapiro–Wilk test was used for normality of variances, as well as Pearson skewness coefficient. Differences were tested by a multivariate ANOVA with feeding time (7 and 15 days of feeding), dietary treatment (CTRL and TRP), stress (stressful condition or not) and sampling time (0-, 4-, 24- and 72-h post-infection) as factors, followed by a *post-hoc* Tukey HSD test, used to identify significant differences amongst groups. Statistical analyses were carried out using IBM SPSS v27.0 and differences were considered statistically significant when *p* ≤ 0.05. A heatmap representing genes’ relative expression was constructed with the free software Heatmapper^32^, using the mean value for each dietary treatment. Pearson’s method was used to calculate the distance metric and relative mRNA levels were hierarchically clustered with the centroid linkage algorithm. To discriminate and classify fish by the existing groups, a multivariate canonical discriminant analysis (DA) was performed on the entire dataset to evaluate linear combinations of the original variables that will best separate the groups (discriminant functions) using Addinsoft XLSTAT 2022 system software. Each discriminant function explains part of the total variance of the dataset and is loaded by variables contributing the most to that variation. Discriminatory effectiveness was assessed by Wilk’s λ test, and the distance between group centroids was measured by squared Mahalanobis distance, and Fisher’s F statistic was applied to infer significance.

## Results

For clarity, results concerning the 7-days feeding period will be presented in "Seven days under the experimental conditions" section, and those obtained after the 15-days feeding period will be presented in "Fifteen days under the experimental conditions" section. Also, the complete set of results related to the relative expression of genes in the hypothalamus, pituitary gland and head-kidney, as well as plasma cortisol levels are available in the Supplementary File (Tables S1–S3).

### Seven days under the experimental conditions

#### Chronic stress-induced changes on the immune status and response

Regarding changes in hypothalamic expression patterns driven by stressful conditions, the expression rates of pro-inflammatory tumour necrosis factor α (*tnfα*), corticotropin-releasing hormone-binding protein (*crhbp*) and δ-opioid receptor (*dor2*) were lower in stressed fish than in unstressed fish, irrespective of other experimental conditions (Table S1). The hierarchical clustering applied to the molecular response on the hypothalamus revealed that unstressed fish fed CTRL at 24- and 72-h post-infection clustered together, based on the expression of the different assessed genes (Fig. 3A).

**Figure 2:**
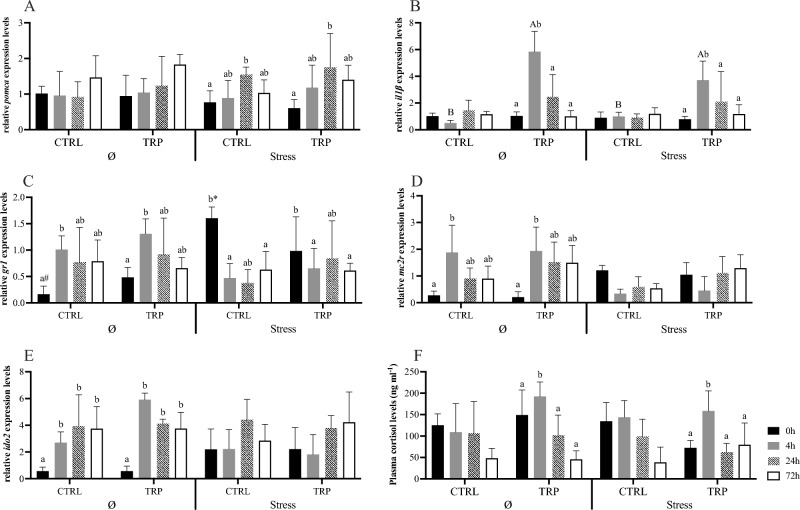
Pituitary gland (**A**,**B**) and head-kidney (**C**–**E**) relative expression of genes related to endocrine-immune processes and plasma cortisol levels (**F**) of European seabass-fed experimental diets (CTRL and TRP) during 7 days under stressful conditions or not (Ø), followed by a bacterial challenge. Pituitary gland: (**A**)—*pomca*, (**B**)—*il1β*. Head-kidney, (**C**)—*gr1*, (**D**)—*mc2r*, (**E**)—*ido2*. Plasma: (F)—cortisol levels. Values are presented as means ± SD (n = 8). Multivariate ANOVA followed by Tukey *post-hoc* test (*p* ≤ 0.05). If interaction was significant, Tukey *post-hoc* test was used to identify differences among treatments. Capital letters stand for significant differences between dietary treatments. Different low-case letters stand for statistically significant differences between sampling times. Different symbols denote significant differences between stress conditions.

**Figure 3:**
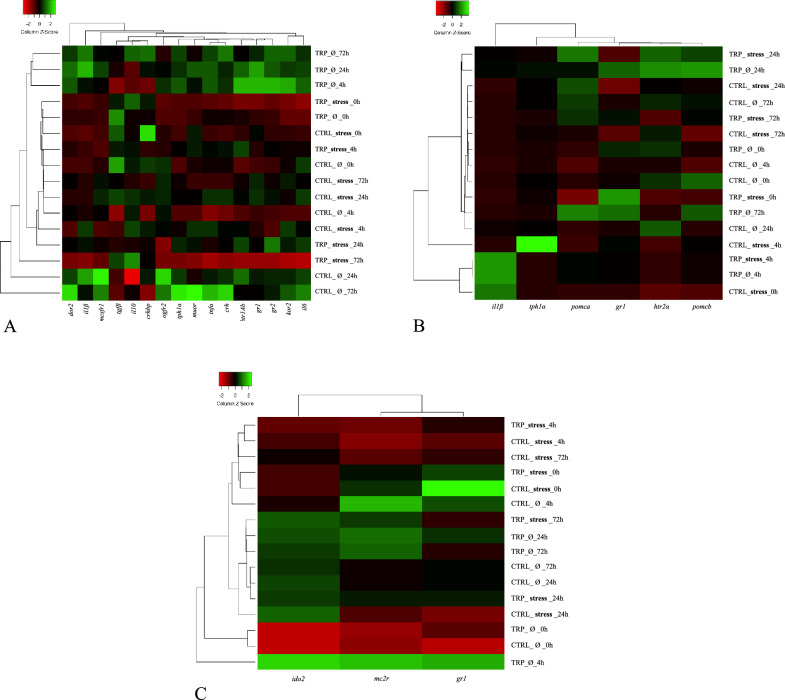
Heatmap of relative mRNA gene expression on the hypothalamus (**A**), pituitary gland (**B**) and head-kidney (**C**) of European seabass fed experimental diets (CTRL and TRP) during 7 under stressful conditions or not (Ø), followed by a bacterial challenge. Lines represent the different dietary treatments and columns represent genes assessed. Pearson’s method was used to calculate the distance metric and relative mRNA levels were hierarchically clustered with the centroid linkage algorithm. Colours represent the intensity of the analysed gene; green more intense; red less intense.

Pro-opiomelanocortin a-like (*pomca*) gene expression in the pituitary was increased at 24 h post-infection in stressed, CTRL-fed fish, while no changes were detected in non-stressed counterparts (Fig. 2A). The heatmap analysis on pituitary gland gene expression resulted in a clear separation of stressed fish fed CTRL before i.p. infection from their infected and unstressed counterparts (Fig. 3B).

In what the head-kidney is concerned, gene expression of glucocorticoid receptor 1 (*gr1*) of CTRL-fed fish before injection was higher in stressed than in non-stressed fish (Fig. 2C). Later, at 4 h after injection, *gr1* was downregulated in stressed fish and remained so in the following sampling points, in contrast to non-stressed fish, where *gr1* expression levels were increased 4 h after i.p. injection. Also, in the head-kidney, melanocortin 2 receptor (*mc2r*) and indoleamine-dioxygenase 2 (*ido2*) were both upregulated 4 h post injection in unstressed fish, whereas no significant changes were observed in fish kept in stressful conditions (Fig. 2D and E, respectively). The low expression intensity of genes in the head-kidney given by heatmap analysis clearly separate unstressed fish fed CTRL at pre-infection time from their post-infection counterparts and from the stressed group (Fig. 3C).

#### Tryptophan modulatory effects in stressful conditions

No significant differences were observed between stressed fish fed TRP and those fed CTRL after 7 days of feeding. In addition, the hierarchical clustering applied to expression patterns in the head-kidney and the hypothalamus revealed that stressed fish fed TRP for 7 days are clustered together with those fed CTRL (Fig. 3C).

#### Tryptophan modulatory effects in inflammatory conditions

With respect to effects of tryptophan dietary supplementation in the course of an immune response (in non-stressful conditions), no significant changes were observed among genes evaluated in the hypothalamus, but the heatmap analysis revealed that upon inflammatory insult (4, 24 and 72 h), unstressed fish fed TRP clustered together and were clearly separated from those fed CTRL sampled at the same time points, based on the expression of the different assessed genes (Fig. 3A).

Considering gene expression patterns in the pituitary gland, *il1β* expression was higher in fish fed TRP than in those fed CTRL at 4 h post-infection (Fig. 2B).

Similar to the hypothalamus, gene expression patterns in the head-kidney were devoid of any significant changes. Nonetheless, the hierarchical clustering showed contrasting expression patterns between TRP-fed fish sampled at 4 h post injection and CTRL-fed fish sampled at the same sampling time (Fig. 3C).

#### Tryptophan modulatory effects during an inflammatory response in stressful conditions

Stressed fish fed CTRL and TRP were used to uncover the TRP modulatory role in combined stress and inflammatory responses. Pituitary *il1β* was upregulated in TRP-fed fish at 4 h post injection, at which point its expression levels were higher than those observed in CTRL-fed fish (Fig. 2B). Its expression returned to basal levels 24 h post injection. In the head-kidney and the hypothalamus, no significant differences were attributed to TRP as both dietary groups showed similar gene expression patterns during the immune response (Tables S1 and S3). Moreover, regarding plasma cortisol, levels peaked at 4 h post infection in fish fed TRP and decreased back to basal levels at 24 h. In contrast, no such differences were found between groups among fish fed CTRL (Fig. 2F).

### Fifteen days under the experimental conditions

#### Chronic stress-induced changes on the immune status and response

**Figure 4:**
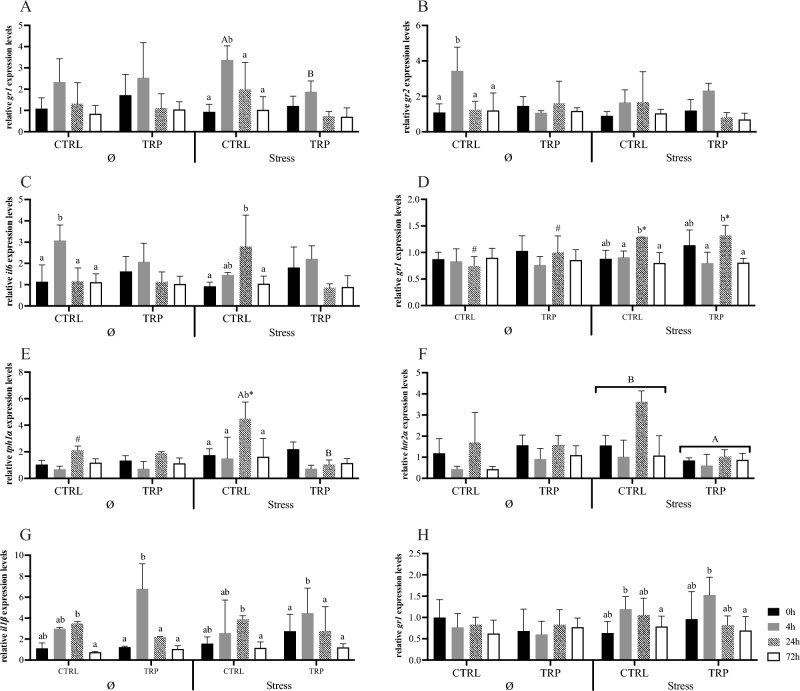
Hypothalamus (**A**–**C**), pituitary gland (**D**–**G**) and head-kidney (**H**) relative expression of genes related to endocrine-immune processes of European seabass fed experimental diets (CTRL and TRP) during 15 days under stressful conditions or not (Ø), followed by a bacterial challenge. Hypothalamus: (**A**)—*gr1*, (**B**)—*gr2* and (**C**)—*il6*. Pituitary gland: (**D**)—*gr1*, (**E**)—*tph1α*, (**F**)—*htr2a*, (**G**)—*il1β*. Head-kidney: (**H**)—*gr1*. Values are presented as means ± SD (n = 8). Multivariate ANOVA followed by Tukey *post-hoc* test (*p* ≤ 0.05). If the interaction was significant, Tukey *post-hoc* test was used to identify differences among treatments. Capital letters stand for significant differences between dietary treatments. Different low-case letters stand for statistically significant differences between sampling times. Different symbols denote significant differences between stress conditions.

**Figure 5:**
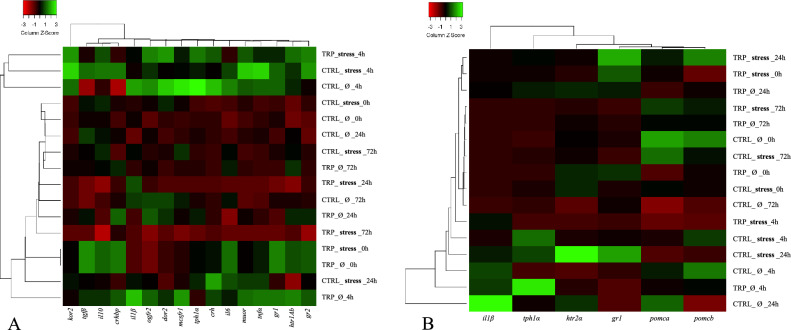
Heatmap of relative mRNA gene expression on the hypothalamus (**A**) and pituitary gland (**B**) of European seabass fed experimental diets (CTRL and TRP) 15 days under stressful conditions or not (Ø), followed by a bacterial challenge. Lines represent the different dietary treatments and columns represent genes assessed. Pearson’s method was used to calculate the distance metric and relative mRNA levels were hierarchically clustered with the centroid linkage algorithm. Colours represent the intensity of the analysed gene; green more intense; red less intense.

Regarding glucocorticoid receptors in the hypothalamus of fish kept in stressful conditions, opposite patterns were recorded for *gr1* and *gr2*. Expression levels of *gr1* peaked at 4 h post-injection in stressed fish fed CTRL, while no changes were detected in unstressed fish (Fig. 4A). On the other hand, *gr2* mRNA levels remained unchanged in stressed fish, while increasing at 4 h post injection in unstressed fish (Fig. 4B). Hypothalamic *il6* gene expression was upregulated at 24 h post injection in stressed fish, and at 4 h post injection in unstressed groups (Fig. 4C). The heatmap analysis showed that hypothalamic gene expression in unstressed and stressed fish-fed CTRL at 4 h post-injection clustered together and clearly separated from the other groups (Fig. 5A). Chronic stressful conditions for 15 days induced pituitary *gr1* gene expression that was upregulated from 4 to 24 h post injection, then returning to basal level at 72 h (Fig. 4D). Plus, at 24 h, *gr1* gene expression was higher in stressed fish than in unstressed fish. Similarly, *tph1α* peaked in stressed fish-fed CTRL at 24 h post-infection and its expression was higher in stressed fish than in the unstressed group (Fig. 4E). No significant differences on mRNA expressions were observed in the head-kidney (Fig. 4H and Table S3).

#### Tryptophan modulatory effects in stressful conditions

Regarding tryptophan-mediated effects in a 15-days stressful setting, no significant changes were observed in any tissue addressed. The hierarchical clustering applied to molecular responses in the hypothalamus and the pituitary gland showed that stressed TRP-fed fish are clearly separated from counterparts fed CTRL (Fig. 5B).

#### Tryptophan modulatory effects in inflammatory conditions

Upon the inflammatory insult by i.p. bacterial injection, hypothalamic *il6* and *gr2* were up-regulated at 4 h post-injection in unstressed fish-fed CTRL, decreasing to basal values after 24 h. Despite the absence of statistical differences with the CTRL group, expression levels remained unchanged for both genes in TRP-fed fish (Fig. 4B,C).

Regarding pituitary *il1β*, while no significant changes were observed in CTRL-fed fish, it significantly peaked in the TRP-fed group at 4 h post-infection (Fig. 4G).

The heatmap analysis in the hypothalamus and the pituitary gland showed different clustering regarding sampling points, as hypothalamic gene expression of unstressed fish-fed CTRL sampled at 4 h post-infection was clearly separated from TRP-fed counterparts, while in the pituitary, a similar separation was observed between CTRL-fed and TRP-fed fish sampled at 24 h post-injection (Fig. 5A,B). No significant differences were observed in the head-kidney (Fig. 4H and Table S3).

#### Tryptophan modulatory effects during an inflammatory response in stressful conditions

Tryptophan dietary supplementation for 15 days in stressful conditions inhibited hypothalamic *gr1* expression after immune stimulation (Fig. 4A). In particular, and contrary to CTRL-fed fish, *gr1* expression was unaltered upon injection in TRP-fed fish, and transcription levels at 4 h were lower than those of their CTRL-fed counterparts. Similarly, hypothalamic *il6* was unresponsive in stressed TRP-fed fish after i.p. injection, while it was gradually increased in CTRL-fed fish, peaking at 24 h post injection (Fig. 4C).

The expression of *tph1α* in the pituitary of stressed fish fed TRP suffered no alterations after injection, in contrast to that of CTRL counterparts, which was induced at 24 h post injection (Fig. 5E). At this timepoint, *tph1α* mRNA levels were lower in stressed TRP-fed fish compared to stressed CTRL-fed fish (Fig. 4E). Also, *htr2a* was transversally downregulated in stressed fish fed TRP, with expression levels lower than those in CTRL-fed fish, irrespective of sampling point (Fig. 4F). In contrast, *il1β* in the pituitary of TRP-fed fish was significantly upregulated from 0 to 4 h post injection (Fig. 4G). No significant differences were observed in gene expression evaluated in the head-kidney (Fig. 4H and Table S3).

The hierarchical clustering applied on hypothalamic gene expression patterns showed that unstressed and stressed fish-fed CTRL and stressed fish-fed TRP at 4 h post-infection are clearly separated from the other sampling points (Fig. 5A). Whereas on the pituitary gland, stressed fish fed TRP after 0 and 24 h post-infection are clearly separated from other sampling points and from CTRL group (Fig. 5).

### Overall correlation among experimental groups

The complete set of results is available in the Supplementary File (Table S4).

**Figure 6:**
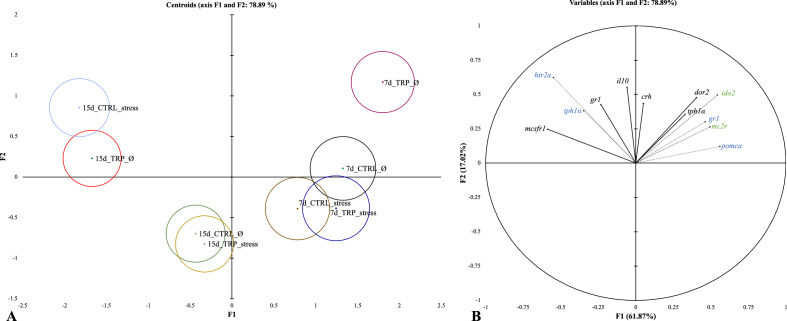
Canonical discriminant analysis of molecular markers of European seabass fed experimental diets (CTRL and TRP) during 7 and 15 days under stressful conditions or not (Ø) and sampled before (0 h) or at 4, 24, and 72 h post bacterial challenge. (**A**) Canonical discriminant scores of each group. Small circle marks represent group centroids. (**B**) Variables/factors correlation (factor loads) for two main discriminant functions (F1 and F2). Hypothalamus (black colour)—*gr1*, *mcsfr1*, *tph1α*, *il10*, *crh* and *dor2*. Pituitary gland (blue colour)—*gr1*, *pomca*, *tph1α*, and *htr2a.* Head-kidney (green colour)—*ido2* and *mc2r*.

**Figure 7:**
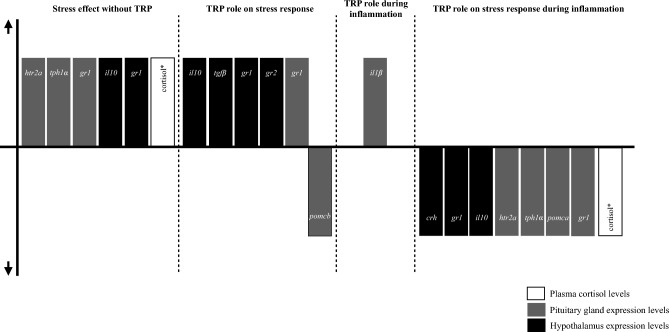
Schematic representation of main gene expression results from the 15 days experiment. *Data published in Machado et al.^28^.

To better understand the role of tryptophan in neuroendocrine–immune interactions and in the mechanisms involved in HPI-axis upon immune stimulation, a canonical discriminant analysis (DA) was performed considering gene expression patterns in the hypothalamus, pituitary gland and head-kidney tissues (Fig. 6). The overall performance of the analysis indicates good discriminatory ability (Wilks λ = 0.142, *p* < 0.0001) with the first two discriminant functions accounting to 78.89% of the total dataset variability (Fig. 7; F1 61.87% and F2 17.02%). Assessing the linear functions of the variables from the analysed tissues, a clear separation by feeding time was observed (Fig. 6A), meaning that fish in both rearing densities and fed both diets for 7 days (that were all different among them) were significantly separated from those fed for 15 days, based on the significant Mahalanobis distance of each group multivariate mean (centroid) (*p* < 0.05). However, within fish fed both diets for 15 days, three separate groups were distinguished: (1) unstressed fish fed CTRL and stressed fish fed TRP; (2) unstressed fish fed TRP and (3) stressed fish fed CTRL (Fig. 6A). The first function, discriminating the groups of fish fed both diets for 7 from those fed for 15 days, was positively loaded by hypothalamus *dor2*, pituitary gland *pomca* and *gr1* and head-kidney *ido2* and *mc2r* (Fig. 6A,B)*,* being negatively loaded by hypothalamus *mcsfr1* and pituitary gland *htr2a* (Fig. 6A,B). The second discriminant function was positively loaded by hypothalamus *gr1* and *il10* and pituitary gland *tph1α* (Fig. 6A,B).

## Discussion

In the present study, the response of stressed, TRP-fed seabass to a bacterial infection was observed to be conditioned by the different contexts.

It is nonetheless useful to first look at the response of stressed fish fed a control, non-supplemented diet, and have a clearer perspective of their neuroendocrine/immune responses before and after an immune challenge. Both stressed and unstressed fish were submitted to an inflammation insult generating immune and neuroendocrine responses and triggering their complex bi-directional network of signalling molecules, receptors, and regulatory mechanisms. In the hypothalamus and pituitary gland of non-injected fish, stress did not significantly alter gene expression, neither after 7 nor after 15 days of stressful conditions. In contrast, in the head-kidney, at the end of 7 days (without any immune stimulation), stressful conditions enhanced *gr1* expression levels. Although GR transcription seems to be much more susceptible to immune signals than to cortisol itself^33^, stressful context for as long as 7 days might have had an impact on regulatory mechanisms (GR) of neuroendocrine effects on immune cells.

Differently, stressful conditions seemed to mostly affect the development of the neuro-endocrine-immune response triggered by i.p. bacterial injection. Interestingly, at the hypothalamus level of stressed fish, a bacterial injection promoted an increase of *gr1* expression, whereas in the absence of stress, the same stimulus induced *gr2*. In the pituitary, too, immune stimulus-triggered *gr1* rise was clearly enhanced by stress, 24 h post injection. As mentioned above, at least as observed in carp (*Cyprinus carpio*), GR’s gene expression is not as susceptible to higher/lower cortisol concentrations, as it is to immune stimulation. Its expression is normally reduced under chronic stress as a regulatory mechanism of stress-induced effects^12^. The observed central increase of both GR’s is here suggested to be brain and pituitary gland’s remaining capacity to respond to an acute stress (immune stimulation) and might indicate some degree of resilience of fish to stressful rearing conditions.

Nonetheless, the fact that one GR was induced in the brain of unstressed fish and another GR in the brain of stressed fish gives strength to Stolte and co-workers’^33^ proposition of a highly sensitive (sensitive to lower levels of cortisol, GR2) and an “insensitive” cortisol receptor (requiring very high levels of cortisol, GR1).

GR activation during a stress response is known to trigger changes at the expression levels of certain genes^34^. Lower *tnfα*, *crhbp* and *dor2* expression in stressed fish from the present study show a clear down-regulatory power of chronic stress on the communication pathways established between the immune and the neuroendocrine systems. In addition, although no differences were detected in expression levels between both groups, central *il6* expression enhancement post injection was delayed in stressed fish, which peak was only at 24 h, later than that of fish kept in control conditions (4 h).

The paralogue pro-opiomelanocortin a-like (*pomca*) is responsible for generating adrenocorticotropic hormone (ACTH)^35^. ACTH, in turn, stimulates the release of cortisol by the interrenal cells in the head-kidney^12,36^. After being fed the CTRL diet for 7 days, stressed fish exhibited an up-regulation of *pomca* at 24 h post-infection, which could have eventually led to higher circulating ACTH and ultimately to an increase in cortisol levels. However, contrary to expectations, such was not the case. Kobayashi et al.^37^ also reported similar findings during fish transfer, observing an increase in mRNA *pomca* levels that was not accompanied by a corresponding rise in plasma cortisol levels. Still, this signalling pathway is further relying in *pomca* effective processing and translation, and on the activation of MC2R by ACTH, for a positive signal for cortisol secretion. And, each of these particular steps is subject to its own regulatory mechanisms. Moreover, POMC not only translates to ACTH, but also to MSH, β-endorphin and β-lipotropin, which also take a part during neuroendocrine responses^31^.

In parallel to this central axis of the neuroendocrine response, serotonergic activity might have been enhanced by stress. Together, the enhanced expression of *gr1* (at both central and head-kidney levels), pituitary *tph1α* and *htr2a* in fish kept at high density illustrate an ongoing neuroendocrine response triggered by the acute inflammatory insult. This difference is also clearly observed in the discriminant analysis, where the two groups, stressed CTRL-fed fish and unstressed CTRL-fed fish are significantly far apart. In particular, there seems to be an enhancement of the serotonergic activity after i.p. injection, and not a classical activation of the HPI-axis with increasing CRH levels. Intra-peritoneal injection (irrespective of its content) is known to trigger central responses in fish, too^38^, with induction of *crh* in the first hours. The absence of a similar enhancement in this study might have been related with regulatory mechanisms (corroborated by *gr1* enhancement) that refrained brain from further stimulating an already activated HPI-axis. Alternatively, an earlier post-injection sampling point (e.g. 1 h) could have detected a missing *crh* peak. Proper assessment of serotonin levels (or even better its metabolite 5-hydroxyindole acetic acid) would more accurately reflect serotonin production rate. Still higher expression rates of this enzyme in the present stressful contexts might indicate a stress-induced enhancement of central serotonergic activity in response to immune stimulation.

At the other hand of the axis—the head-kidney—ACTH receptor MC2R and tryptophan-metabolising enzyme IDO2 upregulation after i.p. injection was not observed in stressed fish, as it was in unstressed counterparts. Immunosuppressive effects have long been attributed to cortisol, as part of its core physiological role as a regulator of inflammatory responses^12^. However, when high concentrations remain for longer periods these effects might compromise immune responses efficiency. Gene transcription of IDO2 is naturally induced upon immune stimulation^39^. The fact that this gene is apparently susceptible to cortisol-mediated downregulation emphasizes the importance of tryptophan metabolic pathways in immune-neuroendocrine network.

As for *mc2r*, downregulation of the ACTH receptor might be a negative feedback mechanism that impairs further cortisol secretion by the head-kidney^40,41^.

In a second analysis of the data, the effects of dietary tryptophan surplus were assessed in stressed fish, in the absence of further immune stimulation. Altogether, hierarchical clustering of molecular patterns in both the pituitary and even more so in the hypothalamus, resulted in a clear separation of stressed groups fed TRP and those fed CTRL after 15 days of feeding. In the pituitary, two genes contributing to this distance were *gr1* and *pomcb*, appearing as highly and poorly expressed in fish fed TRP, respectively. Cortisol receptors GR1 and GR2 were also highly expressed in the hypothalamus of TRP-fed fish, compared to the CTRL group. GR1 mediates cortisol effects in different cells and its downregulation has been considered a feedback regulatory mechanism to high circulating cortisol levels, especially in the brain and pituitary gland^42^. Together, *pomcb* downregulation (potentially lower MSH production), and *gr1* overexpression in fish provided a tryptophan surplus and are one more indication of tryptophan modulatory effect on the normal functioning of neuroendocrine mediators. Previous studies have demonstrated that MSH stimulates cortisol release in teleost fish such as rainbow trout^43^ and tilapia (*Oreochromis mossambicus*)^44^, although its potency is lower compared to ACTH.

Besides changes in the expression of genes directly involved in neuroendocrine responses, the hierarchical clustering analysis also highlighted big contrasts concerning hypothalamic *tgfβ* and *il10* expression rates. Cytokines are constitutively produced in several immunocompetent nervous cells, as previously demonstrated in different teleost fish species^45,46^, where they carry maintenance roles. Cytokines are also able to directly stimulate CRH and ACTH secretion but this role has mostly been attributed to pro-inflammatory cytokines^3^. Further investigation is therefore required to get in-depth knowledge of the outcomes of increased central anti-inflammatory cytokine production (in the absence of immune stimulation). Despite the hierarchical clustering analysis did not separate stressed fish fed both diets, those fed TRP exhibited lower levels of *gr1* and *mc2r* transcripts than those in CTRL-fed fish. Consequently, this resulted in lower plasma cortisol levels. Once again, the downregulation of these genes could be attributed to a feedback mechanism that limits further cortisol secretion by the head-kidney.

To deeply understand the modulatory role of tryptophan during an inflammatory insult, a particular focus was given to unstressed fish fed both CTRL and TRP. A classic inflammation panorama is characterized by an early increase of pro-inflammatory cytokines such as IL1β in the head-kidney, and indeed a robust upregulation of *il1β* was observed in the head-kidney of i.p. injected fish, irrespective of dietary treatment^28^. Tryptophan dietary supplementation did not seem to significantly affect gene expression in the head-kidney during the inflammatory response, but it induced changes at the brain and pituitary gland levels. Peripherally-borne IL1β mediates HPI-axis activation that culminates in different endocrine processes^3^, and it might induce the expression of other proinflammatory cytokines in target cells^47^. Accordingly, IL6 was induced after injection in the hypothalamus of fish fed CTRL, while no such increase was observed in those fed TRP. However, and in contrast, transcription of *il1β* was only mildly (not significantly) induced by bacterial infection in our control group, while it was enhanced in the pituitary of fish fed TRP for both 7 and 15 days, 4 h post infection. Increased peripheral levels of IL1β do not necessarily induce brain’s own IL1β production, as observed in mice by Zhang et al.^48^. These changes in TRP-fed fish suggest a disruptive effect of this amino acid on the brain response to immune stimulation. As no differences were noticed in genes related to serotonin, it is not clear which tryptophan-related mechanisms were engaged leading to these alterations.

A clearer picture of the effects of a dietary tryptophan intervention in stressed fish that is faced with an immune challenge was provided by the discriminant analysis when applied to the whole dataset (including non-infected fish, and both feeding periods). Considering several variables at once, results clearly illustrate how a dietary tryptophan intervention impacts – in opposite ways—several mechanisms involved in immune-neuroendocrine communication pathways.

At a first glance, it clearly shows how the effects of stressful conditions were much more evident if kept for 15 days: group 7d_CTRL_stress clustered with 7d_CTRL_Ø and was distanced from 15d_CTRL_stress. As opposed to fish kept in stressful conditions for only 7 days, those sampled only after 15 days had more typical expression patterns of chronic stress such as the shutdown of neuroendocrine responsiveness (lower pituitary *gr1* and *pomcb*, and lower head-kidney *mc2r*) and higher expression of immune regulatory genes (hypothalamic *il10*).

The same time-dependent effectiveness could be applied to the dietary treatment. Tryptophan dietary supplementation for 15 days (and not for 7 days) induced changes in gene expression that went in opposite direction of their CTRL-counterparts. In particular, once TRP was provided to fish in non-stressful conditions (15d_TRP_Ø), these fish’s molecular patterns resembled those of CTRL-fed fish kept in stressful conditions (15d_CTRL_stressed). In contrast, if provided to fish reared at high densities (15d_TRP_stress), TRP acted as a buffer in the neuroendocrine-immune network, favouring a molecular profile similar to that of CTRL-fed, unstressed fish (15d_CTRL_Ø). These two groups were found almost overlapped, and reflected a profile indicative of homeostatic conditions, with lower expression of markers of neuroendocrine activation (hypothalamic *crh* and *gr1*, pituitary *pomca*, *htr2a*, *tph1α* and *gr1*, and head-kidney *mc2r*), and lower expression of immune signals involved in regulatory pathways (hypothalamic *il10* and head-kidney *ido2*). The attenuation of transcription rates of serotonin-related genes in these groups pointed again to a TRP-led condition that implies changes in serotonergic activity. The abovementioned lessened state of neuroendocrine activation was translated into a general reduction of circulating cortisol levels observed in a parallel approach^28^, and highlights a soothing effect of dietary tryptophan during stressful conditions. These results are therefore in line with previous approaches in rainbow trout^25,26^ and Senegalese sole^29^, where a dietary tryptophan intervention has a clear impact on stressed trout cortisol levels and was efficient in increasing disease resistance of stressed sole against bacterial infection.

Apparently, neither chronic stress nor dietary tryptophan supplementation seemed to affect the expression of most opioid receptors. In turn, the rise of their transcript number after i.p. injection followed by a gradual decrease highlights their importance in an acute stress response, as previously suggested elsewhere^39,49^.

## Conclusions

This study evaluated the potential modulatory effects of tryptophan dietary supplementation in an experimental model reflecting rearing conditions frequently found in aquaculture units – stressful and acute bacterial infection. Taken together, results demonstrate how important is exposition time for both stress- and dietary treatment-induced changes to occur. Not only stress had more relevant effects when applied for 15 days (different profile of neuroendocrine-immune mediators’ engagement upon immune stimulation compared to that of unstressed fish), but also tryptophan-supplemented diet more intensely reversed stress markers when provided for 15 days (as opposed to 7 days) to stressed fish. Moreover, while the head-kidney seems to be primarily susceptible to stress exposure—rather than to tryptophan supplementation—the brain and pituitary gland responses to stress and immune stimulation were clearly modulated by tryptophan dietary intervention.

When tryptophan was included as a dietary supplement, it played a key modulatory role in the HPI axis response to stress stimuli (Fig. 7). This was reflected by a further enhancement of regulatory mechanisms (anti-inflammatory cytokines and glucocorticoid receptors) but also by lower ACTH-precursor transcripts, that ultimately reduced peripheral cortisol levels (parallel approach by Machado et al*.*^28^).

**Figure 8:**
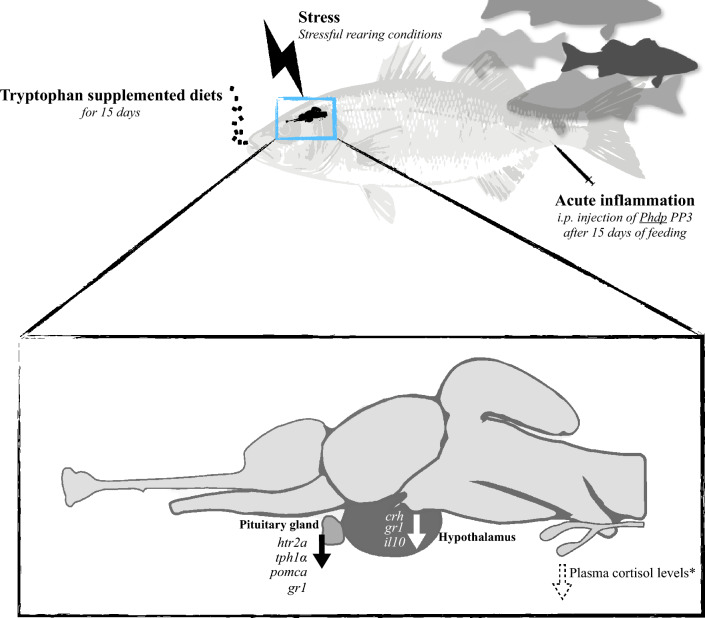
Summary representation of the principal results of the present study. *Data published in Machado et al.^28^.

In a context of combined stress and immune stimulation, dietary supplementation of tryptophan induced notable changes during the immune response at both hypothalamus and pituitary gland levels. Tryptophan surplus inhibited neuroendocrine further activation post infection, decreasing the expression of key signalling hormones (*crh*, *pomca*). Stress-induced immune suppression was similarly counteracted (lower central anti-inflammatory signals and peripheral cortisol levels; Fig. 8). Given the direct relation of tryptophan availability with serotonin pool and the observed changes in the expression of serotonin-related genes (*tph1α* and *htr2a*), it is suggested that the present dietary treatment-induced alterations might be associated to changes in central serotonergic activity.

### Supplementary Information

Supplementary Tables.

## Data Availability

The datasets generated and analysed during the current study are available from the corresponding author on reasonable request.
