# Supplementary material for: Dietary tryptophan intervention counteracts stress-induced transcriptional changes in a teleost fish HPI axis during inflammation
Source: Sci Rep. 2024 Mar 28;14:7354. doi: 10.1038/s41598-024-57761-0 (PMC10978975; doi:10.1038/s41598-024-57761-0)
Supplement: Supplementary file 1 — Supplementary Tables. [file 41598_2024_57761_MOESM1_ESM.pdf]

Dietary tryptophan intervention counteracts stress-induced transcriptional changes in a teleost fish HPI axis during inflammation

Diogo Peixoto[1,2,3]; Inês Carvalho[1,2]; Marina Machado[1]; Cláudia Aragão[4,5], Benjamín Costas[1,2*]; Rita Azeredo[1,2*]

[1] CIIMAR - Centro Interdisciplinar de Investigação Marinha e Ambiental, Matosinhos, Portugal.

[2] ICBAS - Instituto de Ciências Biomédicas Abel Salazar, Universidade do Porto, Porto, Portugal.

[3] Departamento de Biología, Facultad de Ciencias del Mar y Ambientales, Instituto Universitario de Investigación Marina (INMAR), Campus de Excelencia Internacional del Mar (CEIMAR), Universidad de Cádiz, Puerto Real, Spain.

[4] Centro de Ciências do Mar (CCMAR), Faro, Portugal.

[5] Universidade do Algarve, Faro, Portugal.

[*]Corresponding authors – Benjamin Costas | E-mail: bcostas@ciimsr.up.pt
– Rita Azeredo | E-mail: mleme@ciimar.up.pt
Address: Av. General Norton de Matos s/n 4450-208 Matosinhos, Portugal

Supplementary File

Table S1. Hypothalamus relative expression of genes related to neuroendocrine-immune processes of European seabass fed experimental diets (CTRL and TRP) during 7 and 15 days under stressful conditions or not (Ø) and sampled before (0h) or at 4, 24, and 72 hours post bacterial challenge.

	**7 days of feeding**															
	Ø								stress							
	CTRL				TRP				CTRL				TRP			
Relative mRNA expression levels	0h	4h	24h	72h	0h	4h	24h	72h	0h	4h	24h	72h	0h	4h	24h	72h
tnfα	1.3 ± 1.9	1.8 ± 1.2	4.9 ± 1.8	3.4 ± 0.4	1.0 ± 1.4	1.7 ± 1.6	2.7 ± 1.0	1.8 ± 1.4	0.8 ± 0.7	0.8 ± 0.8	3.3 ± 1.9	1.9 ± 0.8	1.0 ± 1.3	0.8 ± 0.5	1.5 ± 1.5	0.6 ± 0.7
tgfβ	1.0 ± 0.3	0.6 ± 0.3	1.0 ± 0.6	0.6 ± 0.3	0.9 ± 0.2	0.6 ± 0.4	0.9 ± 0.4	1.2 ± 0.5	1.4 ± 0.2	1.0 ± 0.5	0.7 ± 0.6	0.8 ± 0.3	1.0 ± 0.3	0.8 ± 0.4	0.9 ± 0.5	0.9 ± 0.4
mcsf1r1	1.1 ± 0.7	0.4 ± 0.2	0.6 ± 0.3	0.5 ± 0.2	1.1 ± 0.3	0.4 ± 0.2	0.7 ± 0.4	0.7 ± 0.2	1.0 ± 0.4	0.5 ± 0.3	0.5 ± 0.4	0.7 ± 0.2	0.8 ± 0.2	0.8 ± 0.6	0.6 ± 0.3	0.9 ± 0.5
htr1aβ	2.5 ± 2.7	1.2 ± 1.6	2.6 ± 2.2	3.9 ± 2.4	1.0 ± 1.1	2.8 ± 2.3	2.6 ± 1.2	2.3 ± 2.0	1.4 ± 1.7	2.0 ± 2.1	4.0 ± 1.6	1.9 ± 1.7	0.6 ± 0.7	1.7 ± 1.8	2.4 ± 2.5	0.3 ± 0.1
il10	0.9 ± 1.1	0.9 ± 1.0	1.7 ± 1.1	1.5 ± 0.1	1.0 ± 1.1	2.8 ± 1.7	2.1 ± 1.0	2.1 ± 1.7	1.1 ± 1.1	0.8 ± 1.0	2.2 ± 1.5	1.2 ± 1.2	0.6 ± 0.5	1.3 ± 1.6	2.3 ± 2.2	0.2 ± 0.1
il6	1.1 ± 0.6	0.6 ± 0.3	1.4 ± 0.8	2.2 ± 1.3	1.1 ± 1.0	1.4 ± 0.7	1.4 ± 0.8	1.8 ± 1.1	0.8 ± 0.4	1.2 ± 1.4	1.8 ± 1.3	0.9 ± 0.5	0.7 ± 0.2	1.2 ± 0.8	1.1 ± 0.6	0.6 ± 0.2
gr1	1.1 ± 0.7	0.6 ± 0.4	1.3 ± 0.5	2.1 ± 1.1	1.1 ± 0.6	1.6 ± 0.8	1.6 ± 1.3	1.5 ± 0.8	0.9 ± 0.4	1.4 ± 1.2	1.7 ± 0.9	0.9 ± 0.7	0.8 ± 0.3	1.2 ± 1.0	1.1 ± 0.5	0.5 ± 0.2
gr2	0.8 ± 0.8	0.8 ± 0.7	2.0 ± 1.5	2.1 ± 1.5	0.9 ± 0.5	2.2 ± 1.1	1.9 ± 1.1	2.1 ± 1.3	0.9 ± 0.6	1.4 ± 1.5	2.2 ± 1.4	1.4 ± 0.8	0.9 ± 0.5	1.5 ± 1.4	1.8 ± 1.1	0.6 ± 0.2
tph1α	1.1 ± 1.7	1.7 ± 1.7	4.1 ± 3.5	2.2 ± 1.0	1.4 ± 1.6	2.2 ± 2.7	3.1 ± 0.8	3.6 ± 3.2	0.9 ± 0.7	1.5 ± 2.2	3.1 ± 1.7	1.4 ± 0.9	0.9 ± 1.1	1.3 ± 1.2	2.3 ± 2.7	0.2 ± 0.1
il1β	0.7 ± 0.5	0.8 ± 0.4	2.3 ± 1.2	1.4 ± 0.2	1.2 ± 1.5	2.8 ± 1.4	2.2 ± 1.7	1.4 ± 0.8	1.1 ± 0.7	1.4 ± 1.5	2.1 ± 0.9	1.3 ± 1.4	0.5 ± 0.3	2.0 ± 1.9	2.0 ± 1.9	0.3 ± 0.1
crh	0.9 ± 1.1	0.9 ± 0.7	1.5 ± 0.7	1.3 ± 0.3	1.0 ± 1.1	2.5 ± 1.5	2.4 ± 0.7	1.5 ± 1.5	1.8 ± 1.5	1.1 ± 1.2	2.2 ± 1.2	1.6 ± 1.4	0.6 ± 0.5	1.1 ± 1.4	1.3 ± 1.1	0.4 ± 0.4
crhbp	1.0 ± 0.2	1.0 ± 0.2	0.5 ± 0.1	0.9 ± 0.3	0.9 ± 0.1	0.8 ± 0.2	0.8 ± 0.2	1.1 ± 0.1	0.9 ± 0.1	1.1 ± 0.2	1.0 ± 0.5	0.9 ± 0.4	1.1 ± 0.2	1.0 ± 0.1	0.9 ± 0.3	1.2 ± 0.4
kor2	2.1 ± 2.4	1.4 ± 1.5	2.6 ± 2.2	3.2 ± 1.9	1.2 ± 1.1	4.1 ± 2.0	2.9 ± 0.6	3.4 ± 2.3	1.7 ± 1.6	2.9 ± 2.4	4.2 ± 2.4	2.5 ± 2.0	0.9 ± 0.6	2.1 ± 1.8	2.5 ± 1.4	0.3 ± 0.2
dor2	1.1 ± 1.6	2.4 ± 2.0	3.6 ± 3.2	6.7 ± 3.5	1.6 ± 1.9	4.0 ± 3.5	4.5 ± 0.8	3.5 ± 2.9	0.9 ± 0.6	0.9 ± 1.0	3.7 ± 0.9	2.3 ± 1.6	1.2 ± 1.7	1.7 ± 2.3	2.4 ± 2.9	0.3 ± 0.2
ogfr2	1.5 ± 2.2	1.5 ± 1.5	2.4 ± 1.5	1.9 ± 0.9	0.7 ± 0.8	1.1 ± 1.0	1.3 ± 0.3	1.2 ± 0.8	1.0 ± 1.5	1.2 ± 1.8	2.1 ± 1.3	1.6 ± 1.6	0.6 ± 1.0	1.0 ± 0.8	0.9 ± 0.5	0.3 ± 0.5
muor	1.2 ± 0.9	0.9 ± 0.5	1.2 ± 0.5	2.9 ± 1.4	1.0 ± 0.6	1.5 ± 0.9	1.9 ± 0.2	1.3 ± 0.6	1.6 ± 1.2	1.7 ± 1.1	1.6 ± 0.6	1.0 ± 0.8	0.9 ± 0.1	1.5 ± 0.6	1.5 ± 0.9	0.5 ± 0.2

Table S1. (Continued).

	7 days of feeding														
**Multifactorial ANOVA**								Diet		Stress		Sampling Time (hours)			
Relative mRNA expression levels	Diet	Stress	Sampling Time	Diet x Stress	Diet x Sampling Time	Stress x Sampling Time	Diet x Stress x Sampling Time	CTRL	TRP	Ø	Stress	0	4	24	72
tnfα	<0.05	<0.05	<0.001	ns	ns	ns	ns	A	B	*	#	a	a	b	a
tgfβ	ns	ns	ns	ns	ns	ns	ns								
mcsf1r1	ns	ns	<0.05	ns	ns	ns	ns					b	a	a	a
htr1aβ	ns	ns	ns	ns	ns	ns	ns								
il10	ns	ns	ns	ns	ns	ns	ns								
il6	ns	ns	ns	ns	ns	ns	ns								
gr1	ns	ns	ns	ns	ns	ns	ns								
gr2	ns	ns	ns	ns	ns	ns	ns								
tph1α	ns	ns	ns	ns	ns	ns	ns								
il1β	ns	ns	ns	ns	ns	ns	ns								
crh	ns	ns	ns	ns	ns	ns	ns								
crhbp	ns	<0.05	<0.05	ns	ns	ns	ns			*	#	a	ab	ab	b
kor2	ns	ns	ns	ns	ns	ns	ns								
dor2	ns	<0.05	<0.05	ns	ns	ns	ns			*	#	a	ab	b	ab
ogfr2	<0.05	ns	ns	ns	ns	ns	ns	A	B						
muor	ns	ns	ns	ns	ns	ns	ns								

Multivariate ANOVA followed by Tukey *post-hoc* test ($p \leq 0.05$). If interaction was significant, Tukey *post-hoc* test was used to identify differences among treatments. Capital letters stand for significant differences between dietary treatments. Different low-case letters stand for statistically significant differences between sampling times. Different symbols denote significant differences between stress conditions. *tnfα* - Tumor necrosis factor-alpha; *tgfβ* - Transforming growth factor-beta; *mcsf1r1* - Macrophage colony-stimulating factor 1 receptor 1; *htr1aβ* - 5-hydroxytryptamine (serotonin) receptor 1A β-like; *il10* - Interleukin 10; *il6* - Interleukin 6; *gr1* - Glucocorticoid receptor 1; *gr2* - Glucocorticoid receptor 2; *tph1α* - Tryptophan 5-hydroxylase-like ; *il1β* - Interleukin 1 beta; *crh* - Corticotropin releasing hormone; *crhbp* - Corticotropin releasing hormone-binding protein; *kor2* - κ-type opioid receptor-like 2; *dor2* - δ-opioid receptor; *ogfr2* - Opioid growth factor receptor 2; *muor* - µ opioid receptor.

Table S1. (Continued).

	15 days of feeding															
	Ø								stress							
	CTRL				TRP				CTRL				TRP			
	0h	4h	24h	72h	0h	4h	24h	72h	0h	4h	24h	72h	0h	4h	24h	72h
Relative mRNA expression levels																
tnfα	1.2 ± 0.6	3.8 ± 1.6	1.1 ± 1.2	1.9 ± 2.2	1.3 ± 0.6	3.0 ± 1.4	1.5 ± 1.1	1.3 ± 1.3	1.5 ± 0.7	1.8 ± 0.3	1.4 ± 1.0	2.1 ± 1.7	1.4 ± 0.9	2.0 ± 0.6	1.0 ± 0.2	0.7 ± 0.4
tgfβ	0.9 ± 0.2	0.6 ± 0.3	1.0 ± 0.1	0.9 ± 0.5	1.3 ± 0.3	1.2 ± 0.5	1.3 ± 0.7	1.1 ± 0.2	0.9 ± 0.4	1.3 ± 0.3	0.9 ± 0.3	0.8 ± 0.2	1.0 ± 0.4	0.9 ± 0.4	0.9 ± 0.2	0.9 ± 0.4
mcsf1r1	1.1 ± 0.4	0.5 ± 0.2	1.3 ± 0.8	0.7 ± 0.6	1.6 ± 0.6	1.3 ± 0.5	1.2 ± 0.6	1.0 ± 0.3	1.2 ± 0.4	1.5 ± 0.4	1.1 ± 0.9	1.1 ± 0.4	0.9 ± 0.3	1.0 ± 0.6	0.7 ± 0.2	0.8 ± 0.3
htr1aβ	1.0 ± 0.3	2.6 ± 1.3	1.2 ± 0.6	1.8 ± 0.6	2.5 ± 1.9	1.9 ± 1.1	0.8 ± 0.3	1.9 ± 0.8	1.2 ± 0.5	2.2 ± 0.9	2.3 ± 1.6	1.7 ± 1.3	2.2 ± 0.7	1.4 ± 0.3	1.0 ± 0.4	1.0 ± 0.5
il10	1.1 ± 0.6	1.5 ± 0.2	1.0 ± 0.6	1.4 ± 0.4	2.0 ± 1.3	2.0 ± 1.0	1.8 ± 1.4	1.3 ± 0.6	1.3 ± 0.3	2.3 ± 1.2	1.5 ± 1.0	1.4 ± 0.9	0.9 ± 0.2	2.2 ± 1.7	1.3 ± 0.7	0.8 ± 0.4
il6	1.1 ± 0.8^{a}	3.1 ± 0.7^{b}	1.2 ± 0.6^{a}	1.1 ± 0.4^{a}	1.6 ± 0.7	2.1 ± 0.9	1.1 ± 0.5	1.0 ± 0.4	0.9 ± 0.2^{a}	1.5 ± 0.1^{ab}	2.8 ± 1.5^{b}	1.0 ± 0.4^{a}	1.8 ± 0.7	2.2 ± 0.6	0.9 ± 0.2	0.9 ± 0.5
gr1	1.9 ± 0.5	2.3 ± 1.1	1.3 ± 1.0	0.9 ± 0.4	1.7 ± 1.0	2.6 ± 1.7	1.1 ± 0.7	1.0 ± 0.4	0.9 ± 0.4^{a}	3.4 ± 0.7^{Bb}	2.0 ± 0.1^{a}	1.0 ± 0.6^{a}	1.2 ± 0.5	1.9 ± 0.5^{A}	0.7 ± 0.2	0.7 ± 0.4
gr2	1.9 ± 0.5^{a}	3.4 ± 1.3^{b}	1.6 ± 0.5^{a}	1.2 ± 1.0^{a}	1.5 ± 0.5	1.1 ± 0.1	1.6 ± 1.3	1.8 ± 0.2	0.9 ± 0.2	1.7 ± 0.7	1.7 ± 1.7	1.0 ± 0.2	1.2 ± 0.6	2.3 ± 0.4	0.8 ± 0.3	0.7 ± 0.4
tph1α	1.3 ± 1.1	2.8 ± 1.1	0.6 ± 0.5	1.9 ± 1.7	0.8 ± 0.6	3.0 ± 2.1	0.9 ± 0.8	1.1 ± 0.8	1.2 ± 0.5	1.3 ± 0.2	1.6 ± 1.2	1.5 ± 1.6	0.7 ± 0.3	1.5 ± 0.4	2.1 ± 2.0	0.8 ± 0.4
il1β	1.1 ± 0.4	1.8 ± 1.2	1.6 ± 1.4	1.5 ± 0.5	2.0 ± 1.7	2.3 ± 2.1	1.8 ± 1.2	1.9 ± 0.9	1.2 ± 0.2	1.9 ± 0.1	0.9 ± 0.5	1.7 ± 1.4	2.1 ± 1.8	2.2 ± 1.8	0.1 ± 0.4	1.3 ± 0.8
crh	1.2 ± 0.8	1.9 ± 0.1	1.0 ± 0.6	1.2 ± 0.9	2.1 ± 1.2	2.0 ± 0.6	0.9 ± 1.0	1.0 ± 0.3	1.0 ± 0.3	1.9 ± 0.6	1.1 ± 0.4	1.2 ± 0.8	0.7 ± 0.1	1.5 ± 0.2	0.7 ± 0.3	0.8 ± 0.4
crhbp	0.9 ± 0.2	0.9 ± 0.2	1.0 ± 0.1	0.8 ± 0.3	1.2 ± 0.3	1.1 ± 0.4	0.8 ± 0.4	1.0 ± 0.2	1.0 ± 0.4	1.2 ± 0.2	0.9 ± 0.5	1.0 ± 0.2	0.8 ± 0.2	1.1 ± 0.4	0.7 ± 0.3	0.6 ± 0.2
kor2	1.1 ± 0.6	4.8 ± 0.5	0.9 ± 0.4	0.8 ± 0.7	2.3 ± 2.3	2.0 ± 0.4	1.7 ± 2.4	1.9 ± 2.1	0.8 ± 0.4	7.2 ± 3.1	2.6 ± 2.6	1.8 ± 2.8	1.9 ± 1.6	5.8 ± 1.5	0.8 ± 0.5	0.4 ± 0.3
dor2	1.1 ± 0.6	3.2 ± 1.5	1.0 ± 0.7	2.1 ± 1.5	1.1 ± 0.5	1.9 ± 1.1	1.9 ± 1.8	1.1 ± 0.9	1.8 ± 0.7	1.4 ± 0.3	0.9 ± 1.1	1.3 ± 1.4	0.8 ± 0.3	2.7 ± 0.9	0.9 ± 0.4	0.8 ± 0.6
ogfr2	1.2 ± 0.6	2.9 ± 0.3	1.7 ± 1.2	2.3 ± 1.5	1.2 ± 0.4	2.6 ± 0.9	2.5 ± 0.9	1.7 ± 1.2	1.7 ± 0.6	2.2 ± 0.3	1.8 ± 0.5	1.5 ± 0.9	1.0 ± 0.5	2.8 ± 0.8	1.4 ± 0.4	0.8 ± 0.5
muor	1.1 ± 0.1	2.6 ± 1.4	1.4 ± 0.7	0.9 ± 0.4	1.7 ± 0.8	2.7 ± 0.8	1.6 ± 1.0	1.4 ± 0.8	1.4 ± 1.0	3.6 ± 0.5	2.3 ± 1.2	1.2 ± 0.5	1.1 ± 0.4	2.5 ± 0.6	0.9 ± 0.3	0.9 ± 0.4

Table S1. (Continued).

15 days of feeding

Relative mRNA expression levels	Diet	Stress	Sampling Time	Diet x Stress	Diet x Sampling Time	Stress x Sampling Time	Diet x Stress x Sampling Time	Sampling Time (hours)				Stress x Sampling Time (hours)								Diet x Sampling Time (hours)							
												Ø				Stress				CTRL				TRP			
								0	4	24	72	0	4	24	72	0	4	24	72	0	4	24	72	0	4	24	72
tnfα	ns	ns	<0.05	ns	ns	ns	ns	a	b	a	a																
tgfβ	ns	ns	ns	ns	ns	ns	ns																				
mcsfr1	ns	ns	ns	ns	ns	ns	ns																				
htr1ab	ns	ns	ns	ns	ns	ns	ns																				
il10	ns	ns	ns	ns	ns	ns	ns																				
il6	ns	ns	<0.001	ns	ns	<0.05	<0.05					a	b	a	a												
gr1	ns	ns	<0.001	ns	<0.05	ns	ns													a	b	a	a				
gr2	ns	ns	<0.001	ns	ns	ns	<0.05																				
tph1α	ns	ns	ns	ns	ns	ns	ns																				
il1β	ns	ns	ns	ns	ns	ns	ns																				
crh	ns	ns	<0.05	ns	ns	ns	ns	a	b	a	a																
crhbp	ns	ns	ns	ns	ns	ns	ns																				
kor2	ns	ns	<0.001	ns	ns	ns	ns	a	b	a	a																
dor2	ns	ns	ns	ns	ns	ns	ns	a	b	a	a																
ogfr2	ns	ns	<0.001	ns	ns	ns	ns	a	b	a	a																
muor	ns	ns	<0.001	ns	ns	ns	ns	a	b	a	a																

Multivariate ANOVA followed by Tukey *post-hoc* test ($p \leq 0.05$). If interaction was significant, Tukey *post-hoc* test was used to identify differences among treatments. Capital letters stand for significant differences between dietary treatments. Different low-case letters stand for statistically significant differences between sampling times. *tnfα* - Tumor necrosis factor-alpha; *tgfβ* - Transforming growth factor-beta; *mcsf1r1* - Macrophage colony-stimulating factor 1 receptor 1; *htr1aβ* - 5-hydroxytryptamine (serotonin) receptor 1A β-like; *il10* - Interleukin 10; *il6* - Interleukin 6; *gr1* - Glucocorticoid receptor 1; *gr2* - Glucocorticoid receptor 2; *tph1α* - Tryptophan 5-hydroxylase-like ; *il1β* - Interleukin 1 beta; *crh* - Corticotropin releasing hormone; *crhbp* - Corticotropin releasing hormone-binding protein; *kor2* - κ-type opioid receptor-like 2; *dor2* - δ-opioid receptor; *ogfr2* - Opioid growth factor receptor 2; *muor* - µ opioid receptor.

Table S2. Pituitary gland relative expression of genes related to neuroendocrine-immune processes of European seabass-fed experimental diets (CTRL and TRP) during 7 and 15 days under stressful conditions or not (Ø) and sampled before (0h) or at 4, 24, and 72 hours post bacterial challenge.

		**7 days of feeding**															
		Ø								stress							
		CTRL				TRP				CTRL				TRP			
		0h	4h	24h	72h	0h	4h	24h	72h	0h	4h	24h	72h	0h	4h	24h	72h
Relative mRNA expression levels	*htr2a*	1.0 ± 0.3	0.5 ± 0.2	0.8 ± 0.7	1.0 ± 0.4	1.0 ± 0.3	0.8 ± 0.2	0.7 ± 0.2	0.7 ± 0.2	0.8 ± 0.4	0.6 ± 0.3	0.9 ± 0.5	1.0 ± 0.3	0.6 ± 0.3	0.7 ± 0.3	0.8 ± 0.2	0.6 ± 0.2
	pomca	1.0 ± 0.2	1.0 ± 0.7	0.9 ± 0.4	1.5 ± 0.6	1.0 ± 0.6	1.0 ± 0.4	1.2 ± 0.8	1.8 ± 0.3	0.8 ± 0.3^{a}	0.9 ± 0.5^{ab}	1.5 ± 0.2^{b}	1.0 ± 0.4^{ab}	0.6 ± 0.2^{a}	1.2 ± 0.6^{ab}	1.7 ± 1^{b}	1.4 ± 0.4^{ab}
	pomcb	1.1 ± 0.5	0.5 ± 0.3	0.7 ± 0.4	0.8 ± 0.6	0.7 ± 0.6	1.1 ± 0.2	1.2 ± 0.7	1.0 ± 0.4	0.7 ± 0.6	1.1 ± 0.2	1.2 ± 0.7	1.0 ± 0.4	0.6 ± 0.3	0.7 ± 0.3	1.0 ± 0.9	0.8 ± 0.2
	gr1	0.9 ± 0.1	0.8 ± 0.2	0.8 ± 0.2	0.9 ± 0.2	1.0 ± 0.3	0.8 ± 0.2	1.0 ± 0.3	0.9 ± 0.2	0.9 ± 0.2	0.9 ± 0.1	1.3 ± 0.0	0.8 ± 0.2	1.1 ± 0.3	0.8 ± 0.2	1.3 ± 0.2	0.8 ± 0.1
	tph1α	1.0 ± 0.3	0.6 ± 0.3	1.0 ± 0.7	1.0 ± 0.5	0.8 ± 0.5	0.8 ± 0.2	1.0 ± 0.5	0.7 ± 0.4	0.8 ± 0.4	0.8 ± 0.2	1.1 ± 0.8	1.0 ± 0.4	0.8 ± 0.4	0.6 ± 0.2	0.9 ± 0.8	0.8 ± 0.4
	il1β	1.0 ± 0.2	0.5 ± 0.2^{B}	1.4 ± 0.8	1.2 ± 0.2	1.0 ± 0.3^{a}	5.8 ± 1.5^{Ab}	2.5 ± 1.7^{a}	1 ± 0.4^{a}	0.9 ± 0.4	1.0 ± 0.3^{A}	0.9 ± 0.3	1.2 ± 0.5	0.8 ± 0.2^{a}	3.7 ± 1.4^{Bb}	2.0 ± 2.3^{a}	1.2 ± 0.7^{a}

Table S2. (continued).

7 days of feeding																							
**Multifactorial ANOVA**								Stress x Sampling Time (hours)								Diet x Sampling Time (hours)							
	Diet	Stress	Sampling Time	Diet x Stress	Diet x Sampling Time	Stress x Sampling Time	Diet x Stress x Sampling Time	Ø				Stress				CTRL				TRP			
								0	4	24	72	0	4	24	72	0	4	24	72	0	4	24	72
Relative mRNA expression																							
htr2a	ns	ns	ns	ns	ns	ns	ns																
pomca	ns	ns	<0.05	ns	ns	<0.05	ns					a	ab	b	ab								
pomcb	ns	ns	ns	ns	ns	ns	ns																
gr1	<0.05	ns	ns	ns	ns	ns	ns																
tph1α	ns	ns	ns	ns	ns	ns	ns																
il1β	<0.001	ns	<0.001	ns	<0.001	ns	ns													a	b	a	a

Multivariate ANOVA followed by Tukey post-hoc test ($p \leq 0.05$). If interaction was significant, Tukey post-hoc test was used to identify differences among treatments. Capital letters stand for significant differences between dietary treatments. Different low-case letters stand for statistically significant differences between sampling times. *htr2a* - 5-hydroxytryptamine (serotonin) receptor 2a; *pomca* - Pro-opiomelanocortin a-like; *pomcb* - Pro-opiomelanocortin b-like; *gr1* - Glucocorticoid receptor 1; *tph1α* - Tryptophan 5-hydroxylase-like; *il1β* - Interleukin 1 beta.

Table S2. (continued).

	15 days of feeding															
	Ø								stress							
	CTRL				TRP				CTRL				TRP			
	0h	4h	24h	72h	0h	4h	24h	72h	0h	4h	24h	72h	0h	4h	24h	72h
Relative mRNA expression levels																
htr2a	1.2 ± 0.7	0.4 ± 0.1	1.7 ± 1.4	0.4 ± 0.1	1.6 ± 0.5	0.9 ± 0.5	1.6 ± 0.5	1.1 ± 0.5	1.6 ± 0.5	1.1 ± 0.8	3.6 ± 0.5	1.1 ± 0.9	0.9 ± 0.1	0.6 ± 0.5	1.0 ± 0.3	0.9 ± 0.3
pomca	1.0 ± 0.5	1.0 ± 0.5	1.1 ± 0.6	1.7 ± 0.5	0.6 ± 0.3	0.7 ± 0.2	0.6 ± 0.4	0.7 ± 0.2	0.7 ± 0.4	0.7± 0.2	0.6 ± 0.4	1.0 ± 0.2	0.7 ± 0.3	0.6 ± 0.2	0.8 ± 0.3	0.9 ± 0.1
pomcb	1.0 ± 0.2	1.0 ± 0.7	0.5 ± 0.2	0.6 ± 0.1	0.7 ± 0.2	0.7 ± 0.2	0.7 ± 0.6	0.7 ± 0.2	0.7 ± 0.1	0.9 ± 0.5	0.6 ± 0.5	0.8 ± 0.5	0.5 ± 0.1	0.5 ± 0.2	1.0 ± 0.6	0.8 ± 0.3
gr1	0.9 ± 0.1	0.8 ± 0.2	$0.8 \pm 0.2^{\#}$	0.9 ± 0.2	1.0 ± 0.3	0.8 ± 0.2	$1.0 \pm 0.3^{\#}$	0.9 ± 0.2	0.9 ± 0.2^{ab}	0.9 ± 0.1^{a}	$1.3 \pm 0.1^{b*}$	0.8 ± 0.2^{a}	1.1 ± 0.3^{ab}	0.8 ± 0.2^{a}	$1.3 \pm 0.2^{b*}$	0.8 ± 0.1^{a}
tph1α	1.0 ± 0.3	0.7 ± 0.2	$2.1 \pm 0.3^{\#}$	1.2 ± 0.3	1.3 ± 0.4	0.7 ± 0.6	1.9 ± 0.1	1.1 ± 0.4	1.8 ± 0.5^{a}	1.5 ± 1.6^{a}	$4.5 \pm 1.2^{Ab*}$	1.6 ± 1.4	2.2 ± 0.5	0.7 ± 0.3	1.1 ± 0.3^{B}	1.2 ± 0.3
il1β	1.0 ± 0.5^{ab}	3.0 ± 0.1^{ab}	3.5 ± 0.1^{b}	0.8 ± 0.1^{a}	1.2 ± 0.1^{a}	6.8 ± 2.4^{b}	2.2 ± 0.1^{a}	1.1 ± 0.3^{a}	1.6 ± 0.6^{ab}	2.6 ± 3.2^{ab}	3.9 ± 0.4^{b}	1.2 ± 0.6^{a}	2.8 ± 1.6^{a}	4.5 ± 2.4^{b}	2.8 ± 2.3^{a}	1.2 ± 0.4^{a}

Table S2. (continued).

15 days of feeding

		Diet	Stress	Sampling Time	Diet x Stress	Diet x Sampling Time	Stress x Sampling Time	Diet x Stress x Sampling Time	Sampling time (hours)				Diet x Stress				Stress x Sampling Time (hours)								Diet x Sampling Time (hours)							
													CTRL		TRP		Ø				Stress				CTRL				TRP			
									0	4	24	72	Ø	Stress	Ø	Stress	0	4	24	72	0	4	24	72	0	4	24	72	0	4	24	72
Relative mRNA	*htr2a*	ns	ns	<0.05	<0.05	ns	ns	ns	a	a	b	a		*		#																
	pomca	ns	ns	ns	ns	ns	ns	ns																								
	pomcb	ns	ns	ns	ns	ns	ns	ns																								
	gr1	ns	<0.05	<0.05	ns	ns	<0.05	ns													ab	a	b	a								
	tph1α	<0.05	<0.05	<0.05	<0.05	<0.05	ns	ns					b	a*		#					ab	a	b	a								
	il1β	ns	ns	<0.05	ns	<0.05	ns	ns																	ab	ab	b	a	a	b	a	a

ultivariate ANOVA followed by Tukey *post-hoc* test ($p \leq 0.05$). If interaction was significant, Tukey *post-hoc* test was used to identify differences among treatments. Capital letters stand for significant differences between dietary :atments. Different low-case letters stand for statistically significant differences between sampling times. Different symbols denote significant differences between stress conditions. *htr2a* - 5-hydroxytryptamine (serotonin) receptor ; *pomca* - Pro-opiomelanocortin a-like; *pomcb* - Pro-opiomelanocortin b-like; *gr1* - Glucocorticoid receptor 1; *tph1α* - Tryptophan 5-hydroxylase-like ; *il1β* - Interleukin 1 beta.

Table S3. Head-kidney relative expression of genes related to neuroendocrine-immune processes and plasma cortisol levels of European seabass-fed experimental diets (CTRL and TRP) during 7 and 15 days under stressful conditions or not (Ø) and sampled before (0h) or at 4, 24, and 72 hours post bacterial challenge.

			Ø							
			CTRL				TRP			
			0h	4h	24h	72h	0h	4h	24h	72h
Relative mRNA expression levels	7 days	*mc2r*	0.3 ± 0.2[a]	1.9 ± 1[b]	0.9 ± 0.4[ab]	0.9 ± 0.5[ab]	0.2 ± 0.2[a]	1.9 ± 0.9[b]	1.5 ± 0.8[ab]	1.5 ± 0.7[ab]
		ido2	0.6 ± 0.4[a]	2.7 ± 0.8[b]	3.0 ± 2.3[b]	3.8 ± 1.7[b]	0.6 ± 0.4[a]	5.9 ± 0.5[b]	4.1 ± 0.3[b]	3.8 ± 1.2[b]
		gr1	0.2 ± 0.2[a#]	1.0 ± 0.3[b]	0.8 ± 0.7[ab]	0.8 ± 0.4[ab]	0.5 ± 0.2[a]	1.3 ± 0.3[b]	0.9 ± 0.7[ab]	0.7 ± 0.2[ab]
	15 days	*gr1*	1.0 ± 0.4	0.8 ± 0.3	0.8 ± 0.2	0.6 ± 0.3	0.7 ± 0.5	0.6 ± 0.3	0.8 ± 0.4	0.8 ± 0.2
Plasma	7 days	Cortisol	125.4 ± 26.3	109 ± 66.8	106.2 ± 74.4	48.2 ± 23.2	149.2 ± 58.4[a]	192.4 ± 33[b]	102 ± 46.9[a]	45.7 ± 19.9[a]

			stress							
			CTRL				TRP			
			0h	4h	24h	72h	0h	4h	24h	72h
Relative mRNA expression levels	7 days	*mc2r*	1.2 ± 0.2	0.3 ± 0.2	0.6 ± 0.4	0.5 ± 0.2	1.0 ± 0.5	0.5 ± 0.5	1.1 ± 0.6	1.3 ± 0.5
		ido2	2.2 ± 1.5	2.2 ± 1.5	4.4 ± 1.5	2.8 ± 1.2	2.2 ± 1.6	1.8 ± 1.5	3.8 ± 1.0	4.2 ± 2.3
		gr1	1.6 ± 0.2b[*]	0.5 ± 0.3[a]	0.4 ± 0.3[ab]	0.6 ± 0.3[a]	1.0 ± 0.7[b]	0.7 ± 0.4[a]	0.8 ± 0.7[ab]	0.6 ± 0.1[a]
	15 days	*gr1*	0.6 ± 0.3[ab]	1.2 ± 0.3[b]	1.1 ± 0.4[ab]	0.8 ± 0.2[a]	1.0 ± 0.7[ab]	1.6 ± 0.4[b]	0.8 ± 0.2[ab]	0.7 ± 0.3[a]
Plasma	7 days	Cortisol	134.6 ± 43.8	143 ± 38.8	99 ± 39	38.6 ± 35.6	72.4 ± 17.3[a]	158.5 ± 46[b]	63.1 ± 19.9[a]	79.7 ± 50.8[a]

Table S3. (Continued).

Multifactorial ANOVA			Diet	Stress	Sampling Time	Diet x Stress	Diet x Sampling Time	Stress x Sampling Time	Diet x Stress x Sampling Time	Stress x Sampling Time (hours)								Diet x Sampling Time (hours)							
										Ø				Stress				CTRL				TRP			
										0	4	24	72	0	4	24	72	0	4	24	72	0	4	24	72
Relative mRNA expression levels	7 days	*mc2r*	<0.05	<0.05	ns	ns	ns	<0.001	ns	a	b	ab	ab												
		ido2	ns	ns	<0.05	ns	ns	<0.05	ns	a	b	b	b												
		gr1	ns	ns	ns	ns	ns	<0.001	<0.001	a	b	ab	ab	b	a	ab	a								
	15 days	*gr1*	ns	<0.05	<0.05	ns	ns	<0.05	ns					ab	b	ab	a								
Plasma	7 days	Cortisol	ns	<0.001	<0.001	ns	<0.05	ns	ns													a	b	a	a

Multivariate ANOVA followed by Tukey *post-hoc* test ($p \leq 0.05$). If interaction was significant, Tukey *post-hoc* test was used to identify differences among treatments. Different low-case letters stand for statistically significant differences between sampling times. Different symbols denote significant differences between stress conditions. *mc2r* - Melanocortin 2 receptor; *ido2* - Indoleamine-dioxygenase 2; *gr1* - Glucocorticoid receptor 1.

Table S4. Canonical discriminant analysis of molecular markers of European seabass fed experimental diets (CTRL and TRP) during 7 and 15 days under stressful conditions or not (Ø) and sampled before and after the bacterial challenge.

Table S4.1. Mahalanobis distances of each group.

	7d_CTRL_Ø	7d_TRP_Ø	7d_CTRL_stress	7d_TRP_stress	15d_CTRL_Ø	15d_TRP_Ø	15d_CTRL_stress	15d_TRP_stress
7d_CTRL_Ø	0.000	2.953	2.720	3.684	4.374	10.711	11.464	5.314
7d_TRP_Ø	2.953	0.000	4.686	4.168	9.184	13.194	13.997	8.863
7d_CTRL_stress	2.720	4.686	0.000	3.516	2.945	7.743	9.577	3.315
7d_TRP_stress	3.684	4.168	3.516	0.000	5.861	10.308	12.261	3.648
15d_CTRL_Ø	4.374	9.184	2.945	5.861	0.000	3.365	5.287	1.470
15d_TRP_Ø	10.711	13.194	7.743	10.308	3.365	0.000	1.632	3.672
15d_CTRL_stress	11.464	13.997	9.577	12.261	5.287	1.632	0.000	5.613
15d_TRP_stress	5.314	8.863	3.315	3.648	1.470	3.672	5.613	0.000

Table S4.2. *p*-value for Fisher distances.

	7d_CTRL_Ø	7d_TRP_Ø	7d_CTRL_stress	7d_TRP_stress	15d_CTRL_Ø	15d_TRP_Ø	15d_CTRL_stress	15d_TRP_stress
7d_CTRL_Ø	1	0.001	0.002	<0.0001	<0.0001	<0.0001	<0.0001	<0.0001
7d_TRP_Ø	0.001	1	<0.0001	<0.0001	<0.0001	<0.0001	<0.0001	<0.0001
7d_CTRL_stress	0.002	<0.0001	1	0.000	0.000	<0.0001	<0.0001	<0.0001
7d_TRP_stress	<0.0001	<0.0001	0.000	1	<0.0001	<0.0001	<0.0001	<0.0001
15d_CTRL_Ø	<0.0001	<0.0001	0.000	<0.0001	1	<0.0001	<0.0001	0.055
15d_TRP_Ø	<0.0001	<0.0001	<0.0001	<0.0001	<0.0001	1	0.026	<0.0001
15d_CTRL_stress	<0.0001	<0.0001	<0.0001	<0.0001	<0.0001	0.026	1	<0.0001
15d_TRP_stress	<0.0001	<0.0001	<0.0001	<0.0001	0.055	<0.0001	<0.0001	1

Table S4.3. Variables correlation/factors.

Tissue		F1	F2	F3	F4	F5	F6	F7
Hypothalamus	*dor2*	0.404	0.478	-0.539	-0.163	-0.426	-0.173	0.096
	tph1α	0.329	0.355	-0.432	0.233	-0.153	-0.524	-0.299
	crh	0.050	0.433	-0.365	0.662	-0.375	-0.090	-0.144
	gr1	-0.232	0.425	-0.174	0.206	-0.041	-0.153	-0.042
	il10	-0.059	0.551	-0.021	0.316	-0.433	-0.263	-0.267
	mcsf1r1	-0.587	0.246	0.280	0.039	-0.257	0.263	0.135
Head-kidney	*ido2*	0.544	0.496	-0.040	0.011	0.222	0.005	0.144
	mc2r	0.494	0.263	0.146	-0.038	-0.350	0.094	-0.296
Pituitary gland	*htr2a*	-0.548	0.623	-0.060	-0.202	0.380	0.001	-0.254
	pomca	0.560	0.122	0.005	0.019	0.101	0.513	-0.295
	gr1	0.463	0.301	0.558	-0.017	0.038	-0.412	0.069
	tph1α	-0.345	0.381	0.079	0.277	0.151	-0.207	0.326

dor2 - δ-opioid receptor; *tph1α* - Tryptophan 5-hydroxylase-like ; *crh* - Corticotropin releasing hormone; *gr1* - Glucocorticoid receptor 1; *il10* - Interleukin 10; *mcsf1r1* - Macrophage colony-stimulating factor 1 receptor 1; *ido2* - Indoleamine-dioxygenase 2; *mc2r* - Melanocortin 2 receptor; *htr2a* - 5-hydroxytryptamine (serotonin) receptor 2a; *pomca* - Pro-opiomelanocortin a-like.
